# **Potential for pulp revascularization in mature anterior teeth with lateral luxation in relation to the patient’s age at the time of injury**—**a retrospective cohort study**

**DOI:** 10.1007/s40368-024-00947-x

**Published:** 2024-10-15

**Authors:** J. S. Henriksen, E. Lauridsen, S. S. Jensen, T. A. Gerds, N. V. Hermann

**Affiliations:** 1 Department of Pediatric Dentistry and Clinical Genetics, School of Dentistry, Faculty of Health Sciences, University of Copenhagen, Copenhagen, Denmark; 2 Department of Oral and Maxillofacial Surgery, Copenhagen University Hospital, Rigshospitalet, Copenhagen, Denmark; 3 Department of Oral Surgery, Research Section for Oral Biology and Immunopathology, School of Dentistry, Faculty of Health Sciences, University of Copenhagen, Copenhagen, Denmark; 4 Department of Biostatistics, Faculty of Health Sciences, University of Copenhagen, Copenhagen, Denmark

**Keywords:** Pulp revascularization, Pulp necrosis, Lateral luxation, Dental traumatic injury

## Abstract

**Purpose:**

The aim of this study was to investigate the potential for pulp revascularization in relation to patient age at the time of injury following luxation injury of mature anterior permanent teeth.

**Materials and methods:**

A total of 93 teeth from 70 patients were included. The patients were divided into subgroups based on their age at the time of the injury. Statistics: the Aalen–Johansen method was used to estimate the risks of pulp canal obliteration (PCO) and pulp necrosis (PN). The absolute 2 year risks of PCO and PN were obtained with cause-specific Cox regression and reported separately for each cohort, standardised to age at injury and degree of repositioning.

**Results:**

For the group younger than 15 years of age, the risk of PN after 12 months was 62.3% [95% CI 44.9; 79.7] in the cohort from 1972 to 1980 and 28.6% [95% CI 4.9; 52.2] in the cohort from 2012 to 2020. For the age group 16–20 years, the risk of PN after 12 months was 66.7 [95% CI 40.0;93.3] in the cohort from 1972 to 1980 and 25% [95% CI 0.0;55.0] in the cohort from 2012 to 2020. For the age group between 21 and 25, the risk of PN after 12 months was 66.7% [95% CI 40.0; 93.3] in the cohort from 1972 to 1980 and 55.6% [95% CI 23.1; 88.0] in the cohort from 2012 to 2020.

**Conclusion:**

There is potential for pulp revascularization in mature anterior teeth with lateral luxation in patients up to 25 years of age. The risk of PN appears to increase with age.

## Introduction

A lateral luxation injury is defined as a traumatic displacement of a tooth in any direction other than axially. This is accompanied by comminution or fracture of the alveolar socket, usually the facial bone (Andreasen et al. 2019). Diagnosis is based upon both clinical and radiographic observations. As a result of the displacement, the pulpal neurovascular supply is partially or completely disrupted. In some areas on the root surface, there will be a separation injury in the periodontal ligament (PDL), and in other areas, the PDL may be compressed. The surrounding soft tissues may also be affected (Andreasen & Andreasen 1985; Andreasen et al. 2019; Bourguignon et al. 2020; Clark & Levin 2019).

Successful healing following lateral luxation involves reorganisation and reestablishment of the PDL fibres and pulpal revascularization and reinnervation. Revascularization can occur in two ways: by ingrowth of new vessels and nerves through the apical foramen or by anastomosis of pre-exiting microvasculature in the pulp, which may lead to survival of the original pulp. Neural regeneration progresses at a slower rate than vascular regeneration (Andreasen et al. 2019; Balic 2018; Clark & Levin 2019).

It is well-documented that revascularization depends on several factors, such as the stage of root development, concomitant fracture injury and the presence of bacteria in the pulp canal (Andreasen et al. 2019).

Healing complications can occur weeks, months or even years after the luxation injury (Andreasen et al. 2019). The timeline of when to expect complications varies, which emphasises the importance of long-term follow-up. The most common complications following lateral luxation include pulp canal obliteration (PCO) and pulp necrosis (PN), whereas ankylosis-related resorption, external infection-related resorption (EIRR) and tooth loss are less frequent (Andreasen & Pedersen 1985; Andreasen et al. 2019; Clark & Levin 2019; Love 1997). This study will focus on the occurrence of PCO and PN.

PCO is a physiological pulp response and is associated with the process of pulpal revascularization after luxation injuries (Andreasen et al. 2019). PCO is characterised by dentin deposition (tertiary dentin formation) within the pulp, leading to partial or total obliteration of the pulp chamber and/or root canal. Signs of PCO are generally observed approximately 1 year after the traumatic injury and most often reach completion by approximately 5 years after the trauma (Andreasen et al. 2019; Spinas et al. 2021). Teeth with incomplete root formation seem more likely to develop PCO than teeth with complete root formation (Andreasen et al. 1987, 2019; Bastos & Côrtes 2018; Spinas et al. 2021). A scoping review from 2021, which included 4 articles reporting a total of 382 lateral luxated teeth, revealed that PCO was diagnosed in 84/382 teeth (51 with open apices and 33 with closed apices) (Spinas et al. 2021).

PN can occur either as ischaemic sterile necrosis (infarction) caused by disruption of the blood supply at the apical foramen or as an infected liquefactive necrosis caused by bacteria. Sterile necrosis is asymptomatic, whereas infected necrosis may cause subjective symptoms (Abbott 2023; Andreasen & Kahler 2015b; Andreasen & Pedersen 1985; Andreasen et al. 2019; Krastl et al. 2022). PN and infection of the root canal system require endodontic intervention.

In the literature, the incidence of PN varies greatly. A systematic review from 2019 revealed that 44.2% of the mature teeth developed PN (Clark & Levin 2019). Whereas a systematic review and meta-analysis from 2024 revealed that prevalence of PN for mature teeth was 58% (Tewari et al. 2024).

In comparison, the prevalence of PN for immature teeth after a lateral luxation injury was found to be 12% by Nitesh et al. (2024) (Tewari et al. 2024) and 17.5% by Clark & Levin (2018) (Clark & Levin 2018).

Laterally luxated teeth with complete root formation (mature teeth) are significantly more likely to develop PN than those with incomplete root formation (immature teeth), as the possibility of pulp revascularization depends on the diameter of the apical foramen (Andreasen & Pedersen 1985; Andreasen et al. 1986, 2019). The maxillary central incisor is expected to fully formed at 12 years of age (Koch et al. 2017; Moorrees et al. 1963). At this stage, the size of the apical foramen is considered to have decreased significantly. However, to our knowledge, relatively few studies have investigated the exact size of the apical foramen for mature teeth. A stereomicroscopic study from 1956 revealed that the average diameter of the major foramen was 0.4 mm for the maxillary incisors and 0.3 mm for the mandibular central incisor (Green 1956). Andreasen et al. reported that teeth with a diameter of less than 1.5 mm in the apical foramen were significantly more likely to undergo PN (Andreasen et al. 1986). Hence, 1.5 mm seems adequate to permit neurovascular ingrowth and thereby enable revascularization. In Andreasen’s study, the apical foramen was measured on two-dimensional (mesiodistally) conventional periapical radiographs. However, the patency of the apical foramen needs to be considered in three dimensions to include the facio-lingual dimension. The study also concluded that even though the width of the apical foramen seems to be a good predictor for the development of PN after an injury, it does not seem likely that a specific diameter would exclude the possibility of pulp survival (Andreasen et al. 1986).

One of the more severe healing complications after a lateral luxation is the development of EIRR. Management of EIRR requires root canal treatment to arrest the penetration of bacteria and bacterial toxins from the root canal to the periodontal membrane through the exposed dentinal tubules. If not managed in a timely manner, EIRR can ultimately lead to tooth loss. (Abbott 2016; Andreasen et al. 2019; Heboyan et al. 2022). A systematic review from 2019 revealed that the risk of EIRR was 8.5% (Clark & Levin 2019), whereas a systematic review and meta-analysis from 2024 revealed the risk of EIRR was 11% (Tewari et al. 2024).

In 2020, the International Association of Dental Traumatology (IADT) revised the guidelines for the management and treatment of traumatic dental injuries. In contrast to the previous edition, early root canal treatment of lateral luxated mature teeth (with a closed apex) is now advocated to prevent the development of EIRR and because the pulp is likely to become necrotic (Bourguignon et al. 2020). Previously, the guidelines were to wait for signs of pulp necrosis before initiating endodontic treatment (Diangelis et al. 2012). However, it is doubtful whether this generalisation of the treatment concept is beneficial for all patients.

Previous studies have reported that survival/restoration of the neurovascular supply (pulp regeneration) is possible in patients with mature laterally luxated permanent teeth (Andreasen et al. 2019; Clark & Levin 2019). Our hypothesis is that children and adolescents will have better healing potential than adults, due to the fact that the apical foramen is wider. We, therefore, wanted to investigate whether there was a correlation between patient age at the time of injury and the development of PN in mature teeth with lateral luxation.

## Aim of the study

The aim of this study was to investigate the potential for pulp revascularization (pulp healing) in relation to patient age at the time of injury following luxation injury of mature anterior permanent teeth.

## Materials and methods

The material included patients treated and followed up at the University Hospital, Denmark, between 1972–1980 and 2012–2020. Two cohorts were included to include as many patients as possible in the study. Patients who fulfilled the following criteria were included in the study:

1. The permanent anterior tooth had suffered a lateral luxation injury, which was defined as an injury as a traumatic displacement of a tooth in any direction other than axially.
2. The stage of root development was defined as mature/apical closure completed (A_c_) based on Moorree’s classification (Moorrees et al. 1963).
3. Tooth-specific clinical information and radiographs from the time of injury and the subsequent controls according to a standardised protocol are presented.
4. The follow-up period was a minimum of 1 year.
5. The tooth had no previous trauma and no concomitant fractures.
6. There was no severe destruction of the crown caused by dental caries or restorations.

The patients seen between 1972 and 1980 followed the standard follow-up programme, which included control visits at 3, 6 weeks, 6 months, 1, and 5 years(Andreasen & Andreasen 1985; Andreasen & Kahler 2015a; Andreasen et al. 2012; Bourguignon et al. 2020). The patients were followed after the mentioned inclusion period. The median follow-up time was 10.8 years.

The patients seen between 2012 and 2020 who followed the standard follow-up programme included controls at 2, 4, and 6–8 weeks; at 6 months; and at 1 and 5 years. After the implementation of the new guidelines beginning in 2020, the patients were also subjected to a 12 week follow-up. In the case of inconclusive signs or doubts about clinical or radiological signs of pathology, the patients were seen for additional control visits (Andreasen & Andreasen 1985; Andreasen & Kahler 2015a; Bourguignon et al. 2020; Diangelis et al. 2012). The median follow-up time was 2.2 years.

In both cohorts, the patients were divided into three subgroups based on their age at the time of injury: younger than 15, 16–20, and 21–25 years of age. There is no significant reason behind the chosen intervals other than our assumption that the apical foramen decreases with increasing age.

At the first follow-up visit after the injury and the initial treatment, the degree of repositioning of the tooth was evaluated. The categories were either complete or incomplete. The evaluation is based on clinical and radiological findings. In those patients with incomplete repositioning, visible displacement was observed on the X-ray at the first follow-up. We suspected that teeth with an incomplete degree of repositioning had less potential for pulp healing than teeth that were repositioned correctly.

The materials were collected in Denmark. The study followed the guidelines of the Declaration of Helsinki 2013, and the material was anonymized and collected over several years. Thus, according to Danish ethical laws, approval from the University Hospital^1^ and an ethical research committee was required.^2^

### Treatment

The patients treated from 1972 to 1980 received both initial treatment and follow-up at the University Hospital.

The patients treated from 2012 to 2020 received initial treatment at the Regional Emergency Clinic, Denmark. The follow-up examination was performed at University Hospital.

The initial treatment followed the international guidelines for the management and treatment of traumatic dental injuries. The treatment included manual repositioning and stabilisation with a flexible splint for 4 weeks (Andreasen & Andreasen 1985; Andreasen & Kahler 2015a; Andreasen et al. 2019; Bourguignon et al. 2020; Diangelis et al. 2012). All the patients seen after the new guidelines from 2020 were informed about the recommended guidelines concerning early root canal treatment of the lateral luxated tooth/teeth and agreed to leave out root canal treatment and instead only perform root canal treatment if there were signs of PN or EIRR (Bourguignon et al. 2020).

### Clinical and radiological registrations

At the time of the injury, the following parameters were registered: sex, patient age, cause of injury, date and time of injury, tooth type, treatment delay, treatment performed, number of injured teeth, displacement of the tooth, discoloration, tenderness to percussion, percussion tone, mobility of the tooth, sensibility, the condition of the supporting tissue, and the presence of any concomitant fractures.

At the follow-up visits, the following parameters were registered: displacement of the tooth, discoloration, tenderness to percussion, mobility of the tooth, sound on percussion, sensibility, the condition of the supporting tissue, the presence of a fistula or abscess, and the presence of any concomitant fractures (Andreasen & Kahler 2015a).

In both cohorts, an electrometric pulp test (EPT) was performed using a dental electronic analogue pulppen (Denlux B1000 Pulppen, Dental Electronic A/S, Ballerup Denmark) placed on the incisal edge of the tooth (Andreasen & Andreasen 1985; Andreasen & Kahler 2015a). The test was performed at the initial examination and at follow-up examinations. The scale ranged from 0 to 18. In addition, a cold test was also performed on the patients in the cohort from 2012–2020 using an Endo-Frost spray.

At the initial examination, periapical radiographs and an occlusal exposure were taken. At the follow-up, only periapical radiographs were taken. Radiographic examination included recording of dislocation of the tooth, the presence of PCO, periapical radiolucency, external and/or internal repair-related resorption, external and/or internal infection-related resorption, or external and/or internal ankyloses. In addition, clinical images were taken at each follow-up to monitor the tooth for any colour change (Andreasen & Kahler 2015a).

### Pulp canal obliteration

PCO was diagnosed if:

- Radiographic signs of obliteration were observed, narrowing in size (partial or total) of the pulp chamber and/or root canal system.
- Yellow discoloration of the crown
- Reduced or absent response to pulp sensibility tests

### Pulp necrosis (cohort from 1972 to 1980)

PN was diagnosed if a minimum of two of the following clinical signs were present:

- Grey discoloration of the crown
- No response to EPT
- Periapical radiolucency

Or if one of following unconditional signs of PN were present:

- Fistula
- Abscess
- EIRR

### Pulp necrosis (cohort from 2012 to 2020)

PN was diagnosed if a minimum of two of the following clinical signs were present:

- Non-reversible grey discoloration of the crown or late grey discoloration of the crown
- No response to pulp sensibility tests (cold and EPT) after 12 months
- Periapical radiolucency

Or if one of following unconditional signs of PN were present:

- Fistula
- Abscess
- EIRR

### External infection-related resorption

EIRR was diagnosed if the following two signs were present:

- Radiographic signs of progressive loss of tooth substance along the root are associated with persistent or progressive radiolucency in the adjacent alveolar bone.
- No response to pulp sensibility tests

All the materials were assessed and calibrated by two trained dentists.

### Statistical methods

The two cohorts were analysed using event history analysis. The time from the date of injury until the PCO, PN, EIRR or last control was calculated, whatever came first. The onset date of PCO or PN was approximated by the middle of the calendar time interval, which ended at the diagnosis of the event and started at the date of the preceding control visit.

The median follow-up time was estimated using the reverse Kaplan‒Meier method. The Aalen–Johansen estimator was used to estimate the risk of PCO, and the Kaplan‒Meier method was used to estimate the risk of PN. The absolute 2-year risks of PCO were estimated using cause-specific Cox regression models, one for the rate of PCO and one for the rate of the competing risk of PN without PCO, adjusted for cohort, age group and degree of repositioning. Cox regression was also used to estimate the absolute 2 year risk of PN. The differences in the standardised average 2 year risks between the cohorts were reported with 95% confidence intervals (Chuang et al. 2001; Ozenne et al. 2020; TA Gerds et al. 2009).

## Results

A total of 93 teeth in 70 patients fulfilled the inclusion criteria. The cohort collected from 1972 to 1980 comprised 62 teeth in 49 patients, and the cohort collected from 2012 to 2020 comprised 31 teeth in 21 patients.

Patient characteristics are shown in Table 1. It is worth noting that the cohort from 1972 to 1980 consisted of twice as many teeth (n = 62) as did the cohort from 2012 to 2020 (*n* = 31). There was a slight predominance of men in the study. Overall, most participants were under 15 years of age (*n* = 41, 58.6%).

**Table 1:** Patient characteristics

Variable		1972–1980 *n* patients (%)	2012–2020 *n* patients (%)	Total *n* patients (%)	*P* value
Sex	Male	31 (63.3)	14 (66.7)	45 (64.3)	
	Female	18 (36.7)	7 (33.3)	25 (35.7)	1.0000
Age group	< 15	31 (63.3)	10 (47.6)	41 (58.6)	
	16–20	10 (20.4)	4 (19.0)	14 (20.0)	
	20–25	8 (16.3)	7 (33.3)	15 (21.4)	0.2709

Our evaluation of the X-rays obtained at the time of trauma after repositioning of the teeth showed that some of the teeth seemed to be incompletely repositioned. In the cohort from 2012 to 2020, as many as 61.3% of the teeth (19 teeth) seemed to be incompletely repositioned (*P* < 0.0001), whereas this percentage was 30.7% (19 teeth) in the cohort from 1972 to 1980 (*P* < 0.0001). Our results showed that the degree of repositioning did not significantly affect the risk of PN or PCO.

### Risk of pulp canal obliteration

Table 2 shows the estimated risks of PCO after 12 months in relation to age groups. The estimated risk of PCO was 23.7% for the age group younger than 15 years, 8.3% for the age group 16–20 years and 0% for the age group 21–25 years in the cohort from 1972 to 1980.

**Table 2:** Absolute risk according to age group estimated 12 months after injury in the two cohorts

Cohort	Age (years)	Number of teeth	Number of events (PCO)	Risk of PCO (%) [95% CI]	Number of events (PN)	Risk of PN (%) [95% CI]
1972–1980	≤15	38	9	23.7 [10.2;37.2]	22	62.3 [44.9;79.7]
1972–1980	16–20	12	1	8.3 [0.0;24.0]	8	66.7 [40.0;93.3]
1972–1980	21–25	12	0	0.0 [0.0;0.0]	8	66.7 [40.0;93.3]
2012–2020	≤15	14	1	7.1 [0.0;20.6]	4	28.6 [4.9;52.2]
2012–2020	16–20	8	1	12.5 [0.0;35.4]	2	25.0 [0.0;55.0]
2012–2020	21–25	9	0	0.0 [0.0;0.0]	5	55.6 [23.1;88.0]

The estimated risk of PCO was 7.1% for the age group younger than 15 years, 12.5% for the age group 16–20 years and 0% for the age group 21–25 years in the cohort from 2012 to 2020.

**Fig. 1:**
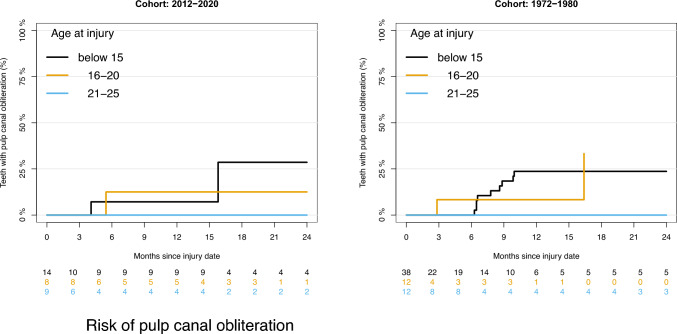
Risk of pulp canal obliteration in relation to age group in the cohorts from 2012 to 2020 and 1972 to 1980 over time

Figure 1 shows the estimated risk of PCO over time in relation to age groups. The graph illustrates a time period of 24 months in total.

In the cohort from 2012 to 2020, the risk of PCO increased for the age group younger than 15 years after 12 months. The risk of PCO is stable for the two other age groups.

In the cohort from 1972 to 1980, none of the patients in the 16–20 years age group were followed for 24 months.

The estimated risk of developing PCO when adjusted for age group and the degree of repositioning was 19.9% after 2 years in the cohort from 2012 to 2020 and 16.9% in the cohort from 1972 to 1980. There were no significant differences in the estimated risk between the two cohorts***.*** This is shown in Table 3.

**Table 3:** Differences in risk adjusted for age group and degree of repositioning

Event	Years	A	Estimate.A (%)	B	Estimate.B (%)	Difference (%)
PCO	2	2012–2020	19.9	1972–1980	16.9	− 3.0 [− 17.8;11.8]
PN	2	2012–2020	40.1	1972–1980	72.2	32.1 [14.7;49.5]

**Fig. 2:**
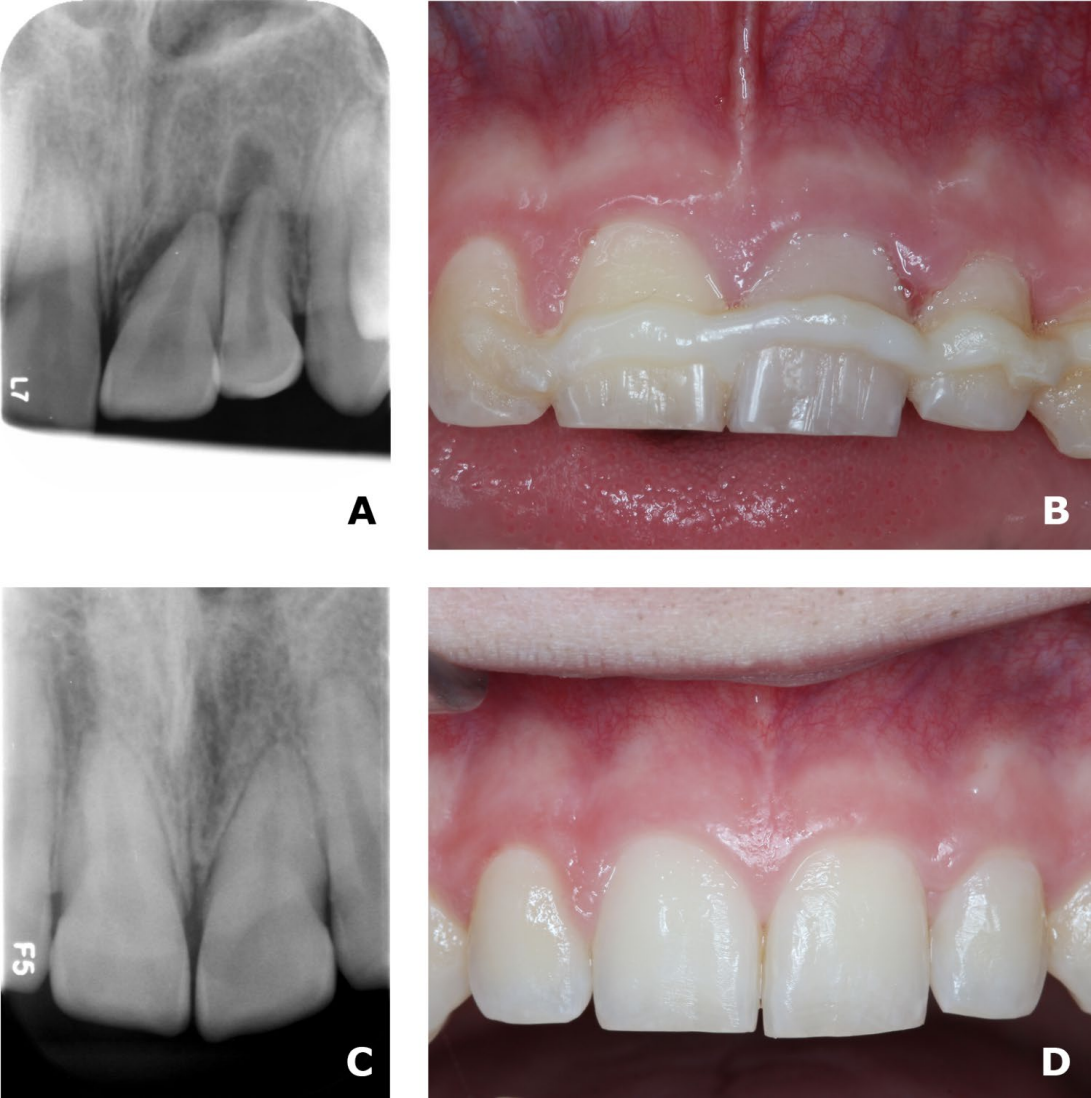
An example of a patient from the cohort from 2012 to 2020. The patient was diagnosed with lateral luxation of 21 and 22. 3**A** Radiographs of 21 and 22 before initial treatment was performed. The displacement of 21 and 22 is clearly observed. 3**B** A clinical photo taken at the 2 week follow-up control. The teeth are stabilised with a splint as recommended by the guidelines. The fixation was performed using a fixator made of Protemp (3 M^™^ Protemp^™^ Plus Temporization Material), which is a composite-based temporization material that allows micromovement of the teeth during the healing phase. Note that 21 has a greyish discoloration of the tooth crown. The discoloration was not there when the initial treatment was performed. 3**C** The radiograph taken at the 2 year follow-up visit. Partial PCO occurs in 21. Both teeth had a positive response to the pulp sensibility test. It was concluded that pulp healing occurred in 21 and 22. 3**D** Clinical photo taken at the 2 year follow-up visit. The discoloration of 21 is now gone

**Fig. 3:**
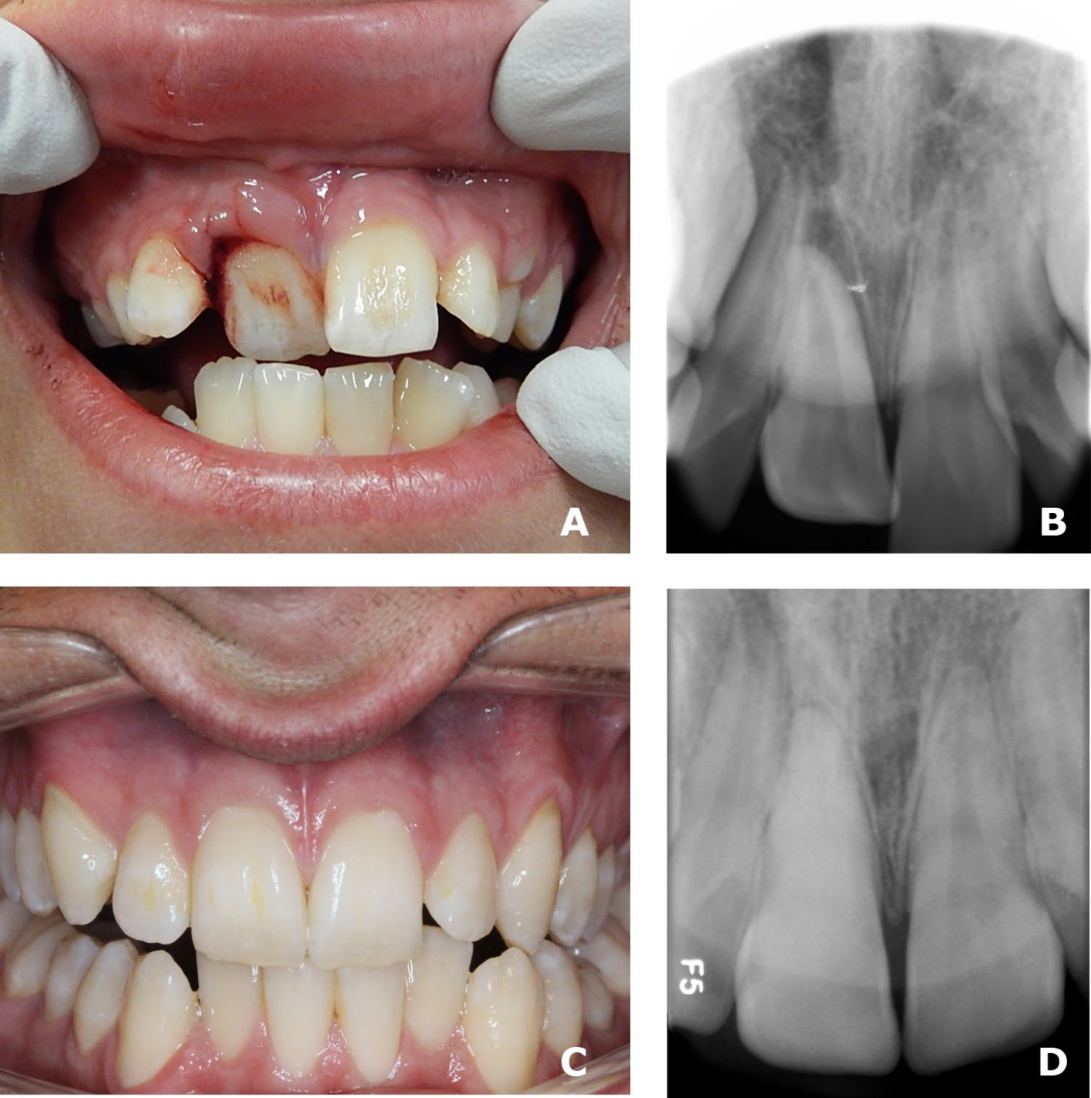
An example of a patient from the cohort from 2012 to 2020. The patient was diagnosed with a lateral luxation of 11. 3**A** A clinical photo, and 3**B** a radiograph before initial treatment was performed. 3**C** Clinical photo and 3**D** radiograph taken at the 1 year follow-up visit. PCO occurs in 11. Note that there is no yellow discoloration of the tooth crown. The tooth had a positive response to the pulp sensibility test. It was concluded that pulp healing occurred in 11

Figures 2 and 3 show examples of two patients from the cohort from 2012 to 2020 in which PCO and pulp healing occurred. Note that in Fig. 2, a temporary discoloration occurs in tooth 21.

Furthermore, the PCO process (the development of PCO) was much faster/pronounced in tooth 11 (Fig. 3) than in tooth 21 (Fig. 2).

### Risk of pulp necrosis

Table 2 shows the estimated risk of PN after 12 months in relation to age groups. The estimated risk of PN after 12 months was 62.3% for those aged younger than 15 years, 66.7% for the age group 16–20 years and 66.7% for the age group 21–25 years in the cohort from 1972 to 1980.

The estimated risk of PN after 12 months was 28.6% for age group younger than 15 years, 25% for the age group 16–20 years and 55.6% for the age group 21–25 years in the cohort from 2012 to 2020.

**Fig. 4:**
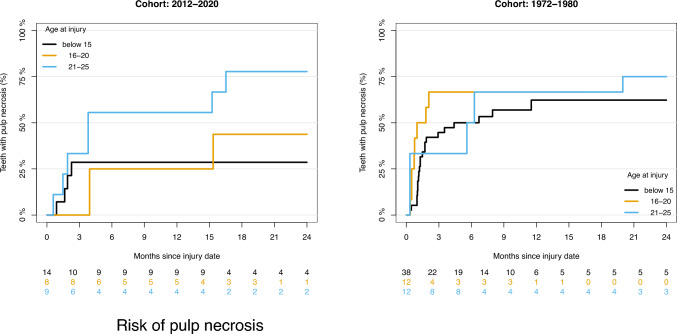
Risk of pulp necrosis in relation to age group in the cohorts from 2012 to 2020 and 1972 to 1980 over time

Figure 4 shows the risk of PN over time in relation to age groups. In the cohort from 2012 to 2020, the results showed that the risk of PN increased with increasing age, as the risk for PN was lowest for age group younger than 15 years, followed by the age group between 16 and 20. The risk for PN was highest for the age group between 21 and 25 years. In the cohort from 1972 to 1980, there was less difference between the risk of PN in the different age groups. Compared with the cohort from 2012 to 2020, the risk for PN in age group younger than 15 years and 16–20 years was significantly greater. For the 21–25-year-old age group, the risk of PN after 24 months was similar in the two cohorts (77.8% vs. 75.0%). It is worth noticing that even though the risk for developing PN is relatively high for individuals aged 21–25 years, there is still an approximately 25% chance for pulp heling.

The estimated risk of developing PN, when adjusted for age group and degree of repositioning, was 40.1% after 2 years in the cohort from 2012 to 2020 and 72.2% in the cohort from 1972 to 1980 (Table 3). The estimated risk of PN in the cohort from 1972 to 1980 was much greater than that in the cohort from 2012 to 2020. There was a significant difference in the estimated risk for PN between the two cohorts.

### Risk of external infection-related resorption

EIRR was observed in 3 out of 93 cases (2.79%) in three different patients. All patients received initial treatment within 1–5 h after the injury, and the degree of repositioning was evaluated to be sufficient. The ages of the patients at the time of the injury were 14, 14 and 20 years.

## Discussion

The results of this study show that the patient’s age at the time of lateral luxation injury should be taken into account when deciding on treatment and seems to question the present guidelines (Bourguignon et al. 2020). In fact, IADT writes in the guidelines that “every effort should be made to preserve the pulp, in both mature and immature teeth”, which may contradict the current recommendation of early endodontic treatment (Bourguignon et al. 2020). Based on the results from this study, it is worth considering whether early endodontic treatment of a lateral luxated mature tooth should be considered only when the patient is older than 25 years of age. The findings suggest that children and adolescents may have better healing potential than adults and that root canal treatment should only be performed if there are signs of PN for patients younger than 25 years of age.

According to a recent article by Abbott (Abbott 2023), ‘preventive’ endodontic management of teeth with lateral luxation injuries should only be provided if there is a concomitant fracture. Hence, endodontic management of a lateral luxation injury with no concomitant fracture should only be considered and commenced when there are definitive signs of pulp necrosis or/and infection of the root canal system (Abbott 2023). A clinical study on this topic would be interesting. Similarly, a study that investigated the healing potential of the pulp based on the patient’s age at the time of lateral luxation injury with concomitant fractures is needed.

Children and adolescents may have better healing potential than adults because the apical foramen is not completely constricted even though the root appears fully developed (with a closed apical foramen) on a periapical radiograph (Andreasen et al. 1986). A future study that compares the size of the apical foramen of lateral luxated teeth in two and three dimensions would be of great interest. A study such as this could establish whether there is any discrepancy regarding the size of the apical foramen between what we see in conventional periapical radiographs and, for example, a cone beam CT scan.

The risk of PCO seems very similar in the two cohorts. More teeth developed PCO in the age group younger than 15 years than in the 16–20 years age group. None of the teeth developed PCO in the 21–25-year-old age group. These findings were similar in both cohorts.

The results show that there is a possibility for developing PCO in patients younger than 20 years of age. Accordingly, there is potential for pulp healing after lateral luxation injury for mature teeth with closed apices in children and adolescents younger than 20 years of age.

The frequency of PN was greater in the cohort from 1972 to 1980. This can be explained by the fact that the observation period before initialling endodontic management of the tooth in the data from 2012 to 2020 was longer than that in the data from 1972 to 1980. For 12 of 49 patients from the 1972–1980 cohort, we currently recommend a longer observation period before endodontic treatment is initiated. In these patients, PN was diagnosed shortly after the injury (within 4 months) based on greyish discoloration of the crown and no response to pulp sensibility tests. Temporary loss of pulp sensibility is a well-known finding during posttraumatic pulp healing (Alghaithy & Qualtrough 2017; Andreasen & Kahler 2015b; Andreasen et al. 2019; Bastos et al. 2014). Regardless of this limitation, pulp sensibility testing should be performed at each appointment to determine if changes occur over time. A combination of cold and electric pulp sensibility tests is preferable due to the limitations of both techniques when used alone. However, this regime for pulp testing was not used in the cohort from 1972 to 1980.

In addition, the discoloration of the tooth can also be transient. Regarding colour changes, it is important to look at when changes in colour occur (Andreasen & Andreasen 1985; Andreasen et al. 2019; Bourguignon et al. 2020; Clark & Levin 2019).

Hence, an accurate diagnosis of the condition of the pulp after TDI is essential to make the correct treatment decision. A correct pulp diagnosis is only possible by combining and assimilating findings (clinical and radiological) and analysing the injury history and injury pattern over time. Regular follow-ups are, therefore, essential (Abbott 2023; Andreasen & Andreasen 1985; Andreasen et al. 2019; Bourguignon et al. 2020; Clark & Levin 2019; Krastl et al. 2022; Lee et al. 2012).

However, it is important to state that even though we would recommend a longer observation time in these 12 patients, the results/outcomes of PN could have been the same. Interestingly, in one patient in whom the dentist diagnosed PN 1.5 months after the injury based on the two clinical signs mentioned above, the pulp actually became vital again. The patient did not receive the recommended endodontic treatment. At the 1-year follow-up, the discoloration had disappeared, and the pulp sensibility test showed a positive response.

As mentioned earlier, the patients treated from 1972 to 1980 received both initial treatment and follow-up at the University Hospital by two specialised dentist in dental trauma. The patients treated from 2012 to 2020 received initial treatment at the Regional Emergency Clinic, Denmark. However, the dentists were trained by the specialists at University Hospital in management of traumatic dental injuries annually. The follow-up examination was performed at University Hospital by three specialised dentist in dental trauma.

The phenomenon of TAB can easily be misdiagnosed as external apical inflammatory resorption. Andreasen et al. first described the process of TAB in 1986 (Andreasen 1986). The exact mechanism of TAB is still not fully understood, but it seems to be linked to the repair process involving the ingrowth of neurovascular supply and elimination of necrotic and damaged pulp tissue after moderate traumatic dental injuries (such as lateral luxation and extrusion injuries) of mature teeth (Andreasen 1986; Andreasen et al. 2019; Cohenca et al. 2003).

TAB, which was misdiagnosed as an apical inflammatory infection, may also be part of the reason why the frequency of PN was higher in the cohort from 1972 to 1980.

In this study, only 3 teeth out of 93 had EIRR (2.79%). Hence, it is worth considering whether early endodontic treatment, which is the recommended guideline, is truly the right approach for all patients. EIRR can be arrested if it is diagnosed and treated in due time. There is no doubt that early detection and management of EIRR is crucial because tooth preservation is unpredictable if large parts of the root are affected/resported. Again, it must be emphasised how important these follow-up visits are (Abbott 2016, 2023; Andreasen et al. 2019; Heboyan et al. 2022). However, early initial EIRR can be difficult to detect, especially on 2D radiographs. Several studies have demonstrated that CBCT scans are more accurate than 2D radiographs at detecting the EIRR at the early stages. Nonetheless, CBCT scans should only be used for signs of EIRR and are not recommended for routine monitoring of teeth at risk of EIRR due to the increased dose of ionising radiation (Patel et al. 2015).

Endodontic treatment is not without consequences for a tooth. Posttreatment disease can occur, and the overall strength of the tooth can decrease, which may lead to a greater risk of subsequent fracture of the tooth (Andreasen et al. 2019; Lee et al. 2012; Ng et al. 2010; Petersson et al. 2016; Tang et al. 2010). The prognosis of a root canal treatment is measured in terms of periapical healing (posttraumatic disease) and tooth survival. This article focuses on tooth survival, which means that the tooth is still present in the mouth. A systematic review of tooth survival following non-surgical root canal treatment revealed that 86–93% of teeth survived more than 2–10 years (Ng et al. 2010). Only a few studies have examined tooth survival after a longer observation time. A study revealed that amongst 889 teeth in 889 patients, 46% of all treated teeth (with primary non-surgical root canal treatment) were still present in the mouth 25 years after trauma (Lee et al. 2012). Two Swedish studies with long observation periods reported that tooth survival of root canal treated teeth was 65% and 71.2% over 20 years (Eckerbom et al. 2007; Petersson et al. 2016). This finding is relevant to keep in mind, especially when treating children and adolescents, since it is expected that most young people will have their teeth for life. Therefore, the risk of EIRR must be weighed against the potential of pulp revascularization to avoid overtreatment (early endodontic treatment/preventive’ (Abbott 2023)) in patients younger than 25 years of age.

The long-term prognosis of traumatised teeth depends on emergency management and how quickly this treatment is provided (Andreasen & Kahler 2015a; Andreasen et al. 2019, 2002). In some parts of the world, patients may not seek treatment until days, weeks, or months after the injury. Lima et al. (2017) investigated the relationship between initial attendance after luxation injuries and the development of EIRR. The results showed that delayed treatment after a luxation injury significantly affects the prognosis and the risk of developing EIRR (Lima et al. 2017). Hence, Soares et al. (2015) reported a high prevalence of EIRR (80.2%) after lateral luxation. The patients in this study underwent initial examinations at 21–90 days, 3–12 months or > 12 months from the date of injury. It is considered very difficult to reposition the tooth correctly after 21 days since wound healing subsequent to the injury has begun, and the possibility of pulp healing must be reduced (Soares et al. 2015).

Likewise, it is important that all dentists maintain their knowledge about traumatic dental injuries so that the level of management is effective, efficient, and correct according to accepted guidelines (Andreasen & Andreasen 1985; Andreasen & Kahler 2015a; Bourguignon et al. 2020; Diangelis et al. 2012).

The recommended guidelines might make sense in areas where patients do not tend to seek immediate dental care after lateral luxation injuries because the risk of developing pulp necrosis and EIRR is significantly greater. However, in countries such as Denmark, where patients show high compliance, meaning that patients seek initial treatment quickly and show up to all the follow-up controls agreed upon, and where the dental health care system is well represented in all regions, we can perhaps be more restrained in our treatment approach. High compliance means that we can monitor the patient closely and, thus, quickly initiate treatment if necessary. In this study, the majority (69.9%) of the patients received initial treatment within 1–5 h after the injury. Only 4.3% of the patients received treatment ≥ 24 h after the injury (*P* value = 0.2956). In addition, all patients were seen at follow-up visits in accordance with applicable international guidelines.

Hence, parameters such as treatment delay, patient compliance, and patient age at the time of injury must be considered to make the best treatment decision for the individual patient.

The retrospective nature of the present study has several limitations. The data were collected during two periods of time (between 1972 and 1980 and between 2012 and 2020). Although the study material consisted of two cohorts, the management of mature teeth with lateral luxation injuries has not changed significantly over the years. Therefore, the inclusion of both cohorts seems to be justifiable. In addition, the data from each designated period were recorded prospectively according to predefined criteria for diagnosis and treatment. Furthermore, clinical and radiographic documentation was also collected. It was possible to verify the original diagnoses due to thorough registration along with clinical photographs and radiographs from the initial examinations. Another important detail that increases the quality of the material is that all radiographs were taken by individual filmholders. This ensured a uniform angle of the X-ray beam each time a radiograph was taken (Andreasen & Andreasen 1985; Andreasen & Kahler 2015a). Thus, the quality of the data can be considered satisfactory. The key differences and similarities between the two cohorts are listed in Table 4. Despite the quality of the data, a limitation is the limited number of patients included. Another limitation of this study is that we do not know the exact day that PCO or PN occurred because the patients were only checked at regular follow-up visits according to the accepted guidelines. Therefore, the onset date of PCO or PN was approximated by the middle of the calendar time interval, which ended at the diagnosis of the event and started at the date of the preceding control visit. To obtain a more accurate picture of the truth (a more exact approximation of when PCO or PN occurred), more frequent follow-up visits could have been carried out.

**Table 4:** Summary of the key differences and similarities between the two cohorts included in the present study

Differences in the cohorts • 2012–2020: Digital radiographs 1972–1980: Conventional film radiographs • 2012–2020: A combination of cold and electric pulp sensibility test was used 1972–1980: Only electric pulp sensibility test • Different observers in 2012–2020 and in 1972–1980 • The observation period for pulp healing before endodontic treatment was initiated was longer in the cohort from 2012 to 2020 than in the cohort from1972 to 1980 • The patients treated from 1972 to 1980 received both initial treatment and follow-up at the University Hospital • The patients treated from 2012 to 2020 received initial treatment at the Regional Emergency Clinic, Denmark. The follow-up examination was performed at University Hospital
Similarities in the cohorts • The management of mature teeth with lateral luxation injuries did not change radically between the two cohorts: The treatment followed the international guidelines for the management and treatment of traumatic dental injuries. The initial treatment included manual repositioning and stabilisation with a flexible splint for 4 weeks • The patients were seen for follow-up visits by few trained specialists in dental trauma (two dentists in the cohort from 1972 to 1980, and three dentists in the cohort from 2012 to 2020) • Use of X-ray holder • Same standardised clinical and radiological examination/registrations performed at each follow-up visits

## Conclusion

Within the limitations of the present retrospective study, it has been shown that there is a potential for pulp revascularization in mature anterior teeth with lateral luxation in patients up to 25 years of age. The risk of pulp necrosis appears to increase with increasing age, whilst the chance of developing pulp canal obliteration was significantly greater for younger patients.

## Footnotes

1 University Hospital approval number: p-2023–14807.

2 Ethical research committee approval number for the project; H-22024452.
